# Pulsed corneal crosslinking in the treatment of Keratoconus: a systematic review and meta-analysis

**DOI:** 10.1007/s00417-024-06622-7

**Published:** 2024-08-31

**Authors:** Maria Qureshi, Stephanie L Watson, Himal Kandel

**Affiliations:** 1 Faculty of Medicine and Health, The University of Sydney, Save Sight Institute, Sydney, NSW Australia; 2 Sydney Eye Hospital, the University of Sydney, Save Sight Institute, South Block, Level 1, 8 Macquarie Street, NSW 2000 Sydney, Australia

**Keywords:** Pulsed, Continuous, Corneal collagen cross-linking, Cross-linking, Keratoconus, Keratometry, Riboflavin UV-A treatment, Efficacy

## Abstract

**Purpose:**

Corneal crosslinking (CXL) procedures are the treatment of choice in halting progressive corneal ectasia and preserving visual acuity due to keratoconus. Pulsed crosslinking (P-CXL) was developed using intermittent pulsing ultraviolet (UV) light to mitigate the depletion of oxygen levels that occurs with continuous UV exposure in standard crosslinking protocols (C-CXL). This study aimed to explore the use of P-CXL in the treatment of keratoconus and determine whether the availability of oxygen in P-CXL carries superior efficacy outcomes as an alternative to C-CXL modalities.

**Methods:**

This review was undertaken in accordance with PRISMA guidelines. A search of several databases conducted with two separate reviewers resulted in 29 papers meeting inclusion criteria for the review, 14 selected for meta-analysis. Primary outcomes assessed by the included papers included maximum keratometry (Kmax), corrected and uncorrected distance visual acuity (CDVA, UDVA), and secondary outcomes included central corneal thickness (CCT), endothelial cell count and demarcation line. Statistical analyses were carried out on Review Manager 5.4 and the meta-analysis employed a random-effects model, which estimated the weighted effect size of raw means using inverse variance weights.

**Results:**

At 12 months P-CXL showed statistically significant reductions in Kmax (-0.75 D; *p* < 0.001) and improvement in CDVA (-0.10 logMAR; *p* < 0.001) compared to baseline. The meta-analysis of comparative studies determined that mean differences in Kmax, CDVA, UDVA, Kmean and CCT after 12 months were not statistically significant between pulsed and continuous crosslinking groups.

**Conclusions:**

Overall, P-CXL is effective in improving visual acuity and keratometry outcomes in keratoconus. The meta-analysis did not show a statistically significant difference in Kmax and CDVA between P-CXL and C-CXL, indicating a non-inferiority of P-CXL. However, findings of the meta-analysis are limited by the fact that different energy levels and exposure times were used for P-CXL in comparison to C-CXL in some studies, making it unsuitable to determine whether the efficacy of CXL is improved by the use of pulsed light.

**Key messages:**

***What is Known***

• Pulsed crosslinking (P-CXL) uses intermittent UV light to prevent oxygen depletion when using higher energy protocols, unlike continuous UV exposure in standard continuous crosslinking (C-CXL).

• This should theoretically enhance the efficacy of the treatment by maintaining higher oxygen levels that are crucial to the cross-linking process.

• There are no systematic reviews or meta-analyses directly comparing the efficacy or safety of P-CXL to C-CXL.

***What is New***

• Meta-analysis revealed differences in keratometry between P-CXL and C-CXL groups with equivalent fluence (7.2 J/cm^2^) at 12 months were not statistically significant (Kmax -0.04 dioptres; *p* = 0.84).

• Meta-analysis revealed differences in visual acuity between P-CXL and C-CXL groups with equivalent fluence (7.2 J/cm^2^) at 12 months were not statistically significant (CDVA -0.01 logMAR letters; *p* = 0.57).

• The use of intermittent pulsing in higher energy CXL protocols renders statistically similar outcomes as continuous light exposure at equivalent fluence (7.2 J/cm^2^)

**Supplementary Information:**

The online version contains supplementary material available at 10.1007/s00417-024-06622-7.

## Introduction

 Keratoconus is an ocular condition that characteristically involves progressive thinning of the cornea and weakening collagen fibers [1] of the corneal stroma. This leads to corneal ectasia that induces astigmatism and reduces visual acuity [2] overall lowering a patient’s quality of life [3–7]. If progression of the disease is reliably detected, corneal crosslinking (CXL) procedures are the treatment of choice for halting further progression and eliminating the need for corneal transplantation [8]. CXL involves the topical administration of Vitamin B2 (riboflavin) to the treated eye followed by exposure to ultraviolet A (UV-A) light. The combination of these maneuvers results in a photochemical reaction that enables covalent cross-linking between corneal stromal collagen fibres and increases overall structural rigidity [9]. Various protocols have been used for the CXL procedure to improve outcomes from the procedure and reduce treatment duration.

The Dresden Protocol for CXL consists of epithelial removal to allow for adequate penetration of 0.1% topical riboflavin administered for 30 minutes followed by UV-A exposure at 3 mW/cm^2^ for 30 minutes, resulting in a total fluence of 5.4 J/cm^2^. The Bunsen-Roscoe Law of Reciprocity suggested that keeping the fluence constant would allow the clinician to make adjustments to UV-A irradiance or total exposure time while achieving the same results [10].To reduce patient discomfort, continuous accelerated protocols were developed that shorten total procedural time by increasing the UV-A irradiance based on the Bunsen-Roscoe Law. In chemical kinetic models, continuous exposure to UV-A leads to total oxygen depletion in the corneal stroma within 10–15 seconds. The presence of oxygen theoretically improves CXL by allowing the formation of singlet oxygen (a reactive oxygen species) that acts on proteoglycan proteins within corneal tissue to drive the production of cross-linked bonds [11]. This has been suggested to be a superior method of CXL to the radical ions produced under anaerobic conditions [12]. Pulsed crosslinking (P-CXL) was developed using a pattern of intermittent pulsing UV light (typically one second on/off) to mitigate the depletion of oxygen levels that occurs with continuous UV-A exposure in standard protocols. Accelerated CXL protocols with pulsed UV-A energies delivered between 9 and 30 mW/cm^2^ may create optimal clinical workflow and patient compliance via a reduced treatment time of 20–25 minutes [13].

Systematic reviews conducted to assess the efficacy of CXL have only provided limited insights into P-CXL as an emerging option in the treatment of progressive keratoconus. The primary objective of this systematic review was to investigate the effectiveness of P-CXL in treating keratoconus and to determine whether it exhibits superior efficacy outcomes compared to the traditional continuous crosslinking (C-CXL) approach. Our intention was to conduct a comprehensive systematic review and meta-analysis that could provide valuable guidance to clinicians in selecting appropriate CXL modalities for their patients. Furthermore, the findings from this review could prove beneficial to clinical trials focused on the development of new CXL protocols.

## Methods

This systematic review was conducted as per Preferred Reporting Items for Systematic Reviews and Meta-Analyses (PRISMA) statement guidelines [14]. A search strategy was developed to include all studies assessing the efficacy of P-CXL in comparison to C-CXL (See Appendix 1). Databases analysed include Medline, Embase, Pubmed, Scopus and Cochrane. Clinical Trials.gov was also searched for any unpublished data. All searches were carried out in May 2023.

### Eligibility criteria for considering studies for review

#### Inclusion Criteria

Prospective or retrospective studies which included patients with keratoconus that had undergone P-CXL and had not received any previous treatment in the affected eye were included. Included studies must have had one or more of the following outcome parameters – visual acuity (corrected or uncorrected), corneal curvature measures (maximum keratometry, mean keratometry, steep keratometry, thinnest pachymetry, central corneal thickness), endothelial morphometry (endothelial cell count) or demarcation line. Studies including 10 or more eyes in patient sample sizes of 10 or more with at least a 3 month follow-up were included in the systematic review. Meta-analysis was conducted on studies with a 12 month follow up and a consistent 7.2 J/cm^2^ fluence level in the pulsed groups.

#### Exclusion Criteria

Studies including patient cohorts that had concurrent ocular conditions or any previous treatments in the affected eye were excluded. Ex-vivo and animal studies were also excluded. Studies not conducted in English were excluded. Studies that looked at fluence levels outside of either 5.4–7.2 J/cm^2^ were considered non-comparable and therefore excluded from the meta-analysis. Studies where the P-CXL procedure was modified or customised (either delivered with topographical guidance, iontophoresis or supplemented with oxygen) were excluded from the meta-analysis but otherwise included for discussion in the review.

### Study selection and data extraction

Literature searching was conducted and retrieved articles were then exported and excluded or included by 2 separate reviewers. Systematic Review management software Covidence was used to remove duplicates, screen abstracts and conduct full-text reviews. Disagreements were cleared by a third reviewer. Data extracted included: study ID (author, year of publication), country, demographic of each cohort, and study characteristics. Primary outcomes included changes in maximum keratometry (Kmax), corrected and uncorrected distance visual acuity (CDVA, UDVA). Secondary outcomes included changes in mean and steep keratometry (Kmean and K2 respectively), central corneal thickness (CCT), endothelial cell count and demarcation line.

For single arm studies analyses were made using generic inverse variance [15] comparing baseline and 12 month values to compare overall mean difference between studies.

### Data synthesis and analysis

For each outcome variable in the meta-analysis, a calculation of differences in paired means was carried out for each included study. In cases where standard deviation (SD) data were not provided, they were derived from other statistical values such as p values, confidence intervals (CIs), and standard errors. The meta-analysis employed a random-effects model, which estimated the weighted effect size of raw means using inverse variance weights. The I^2^ statistic was employed to assess potential heterogeneity among the studies. Statistical and meta-analysis were conducted using Review Manager (version 5.4), an open-source desktop tool developed by Cochrane Collaborations. A two tailed p-value lower than 0.05 was considered to indicate statistical significance.

## Results

**Fig. 1:**
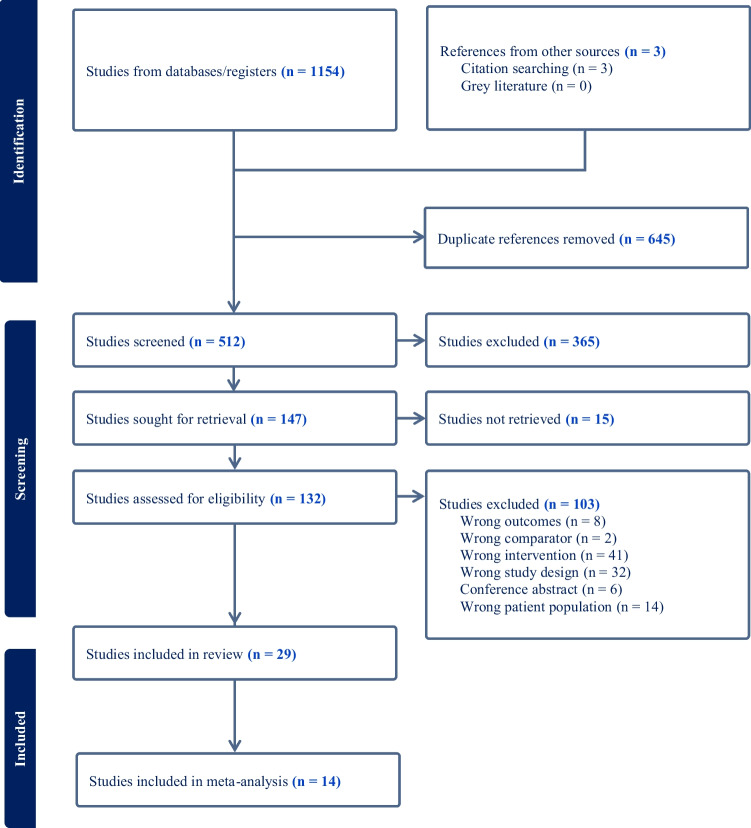
Preferred Reporting Items for Systematic Reviews and Meta-Analyses (PRISMA) flowchart showing the search process to identify relevant articles for inclusion

Of a total 1154 publication titles yielded by the initial search, 29 were selected for the systematic review with 14 of these further included for the meta-analysis (Fig. 1). All studies were either prospective or retrospective studies and there were no randomised control trials. Of the 14 studies in the meta-analysis, five were dual arm and included data sets directly comparing P-CXL and C-CXL and the remaining nine studies were either single or dual arm but lacked C-CXL controls. The study selection process can be found in supplemental data (Online Resource 1).

Characteristics of included studies can be found in Table 1. Reviewed studies were published between 2014 and 2023, including a total of 2866 eyes (1817 of which underwent P-CXL and 1049 underwent C-CXL). Eight studies explored transepithelial methods of CXL while the remainder opted for complete epithelial debridement prior to chosen CXL procedures. Twenty-four studies opted for UV-A energies with corresponding duration of exposure times calculated such that patients in pulsed groups received a total fluence of either 5.4–7.2 J/cm^2^. The few studies with variable fluence had non-uniform irradiance delivery, meaning different fluences were delivered asymmetrically throughout the cornea. The studies were conducted in a variety of countries, providing an international dataset with varied ethnic groups.

**Table 1:** Characteristics of included studies for the systematic review and meta-analysis of pulsed vs. continuous crosslinking

Study	Country	Eyes (*n*)	Study Design	Epithelium (on/off)	Study Arms (*n*)	Total Fluence Delivered (J/cm2)	UV-A Energy (mW)	Duration (mins)	Outcomes Assessed	Follow Up (months)
Abdel-Radi 2023 [16]	Egypt	45	Prospective Case Series	off	1	7.2	30 [P]	8	D, E	3, 6
Artieda 2020 [17]	Spain	62	Retrospective Case Series	off	1	7.2	30 [P]	8	A, K, Pa, R V	12
Belviranli 2020 [17]	Turkey	30	Retrospective Case Series	off	1	5.4	30 [P]	6	K, P, T, V	6, 12, 24
Bohm 2019 [18]	Germany	12	Retrospective Case Series	off	1	7.2	30 [P]	8	K, Pa, V	3
Cronin 2022 [19]	Australia	53	Retrospective Case Series	on	1	10	30 [P]	11	D, K, Pa, R V	1, 3, 6, 12
Cassagne 2017 [20]	France	60	Prospective Non-randomised Trial	off	2	variable	variable	variable	D, K, V	12
Gaafar 2021 [21]	Egypt	72	Retrospective Case Series	off	1	7.2	30 [P]	8	K, Pa	12, 24, 36
Gore 2021 [22]	England	1192	Prospective Case Series	off	2	7.2	30 [P]	8	E, K, Pa, R, V	6, 12, 24
Hernandez 2019 [23]	Mexico	50	Retrospective Cohort Study	off	2	7.2	30 [P] 45 [P]	8 5.3	E, K, Pa, V	1, 3, 6, 12
Jiang 2017 [24]	China	72	Prospective Cohort Study	off	2	7.2 5.4	30 [P] 3 [C]	8 30	D, E, K, Pa, R, V	1, 3, 6, 12
Kang 2021 [25]	Korea	45	Retrospective Cohort Study	off	2	7.2	30 [P] 30 [C]	8 4	D, K, Pa, V	12
Marafon 2020 [26]	Brazil	113	Retrospective Cohort Study	off	2	7.2 5.4	30 [P] 3 [C]	8 30	E, K, V	6
Matthys 2021 [27]	France	34	Prospective Cohort Study	on	1	20	30 [P]	11	D, K, V	1, 3, 6, 12
Mazzotta 2014 [28]	Italy	20	Prospective Case Series	off	2	7.2	30 [P] 30 [C]	8 4	A, D, E, K, V	1, 3, 6, 12
Mazzotta 2017 [29]	Italy	132	Prospective Case Series	off	1	5.4	15 [P]	12	A, D, K, Pa V	1, 3, 6, 12, 24
Mazzotta 2020 [30]	Italy	27	Prospective Case Series	on	1	7.2	30 [P]	8	D, E, K, V	6
Mazzotta 2022 [31]	Italy	40	Prospective Cohort Study	on	1	7.2	18 [P]	13	A, D, K, Pa, V	1, 3, 6, 12, 24, 36
Moramarco 2015 [32]	Italy	70	Retrospective Case Series	off	2	7.2	30 [P] 30 [C]	8 4	D	1, 3, 6
Nordstrom 2017 [33]	Sweden	50	Randomised Control Trial	off	2	variable	variable	variable	E, K, R, V	1, 3, 6, 12
Peyman 2016 [34]	Iran	70	Prospective Case Series	off	2	7.2	30 [P] 30 [C]	8 4	D	3
Sachdev 2021 [35]	India	64	Prospective Case Series	off	2	variable	variable	variable	A, E, R, T, V	1, 6, 12
Shajari 2018 [36]	Germany	58	Retrospective Case Series	off	2	5.4, 7.2	30 [P] 3 [C]	8 30	K, Pa	12
Sun 2018 [37]	China	26	Prospective Case series	on	1	7.2	45 [P]	5.3	E, K, Pa, R, V	1, 3, 6, 12
Tian 2020 [38]	China	53	Retrospective Case Series	on	1	7.2	45 [P]	5.3	K, Pa, R, V	1, 6, 12, 36
Toker 2017 [39]	Turkey	134	Retrospective Case Series	off	2	7.2	30 [P] 30 [C]	8 4	K, Pa, R, V	12
Zhang 2020 [40]	China	42	Retrospective Case Series	on	1	7.2	45 [P]	5.3	E, K, Pa, R, V	1, 3, 6, 12, 24, 36, 48
Ziaei 2019 [41]	New Zealand	40	Prospective Case Series	on	1	7.2	45 [P]	5.3	K, R, T, V	1, 3, 6, 12, 24
Ziaei 2020 [42]	New Zealand	120	Prospective Case Series	off	2	7.2	30 [P] 30 [C]	8 4	K, Pa, R, T, V	1, 3, 6, 12, 24
Ziaei, Gokul 2019 [43]	New Zealand	80	Prospective Case Series	off	2	7.2	30 [P] 30 [C]	8 4	K, R, V	1, 3, 6, 12, 24

Key – *P= *pulsed, *C= *continuous, *A= *aberrometric outcomes, *D= *demarcation line, *E= *endothelial morphometry, *K= *keratometry, *Pa= *pachymetry, *T= *tomographic outcomes, *V= *visual acuity, *R= *refractive outcomes

### Quality assessment

For comparative cohort and case control studies a modified version of the Newcastle-Ottawa Scale [44] was used for assessment of quality and risk of bias in non-randomised settings. This model comprises of domains covering selection, comparability and outcome/exposure between cases, controls and cohorts. Of the five comparative studies, Jiang [24] and Shajari [36] received ratings of seven stars (of a possible nine) rendering them ‘good studies’ while the remaining scored six stars or ‘satisfactory studies’. For non-comparative case series, the quality was assessed using criteria developed by Murad et al [45] covering domains including ascertainment, causality and reporting. In this category, no study received less than a ‘Good’ score, indicating an overall strong quality of studies captured within the meta-analysis despite the lack of randomised control trials (Online Resource 5). Quality assessment was carried out by two separate reviewers. Publication bias assessment via funnel plot was not conducted due to the limited number of studies.

## Effectiveness of P-CXL in Keratoconus

### Primary outcomes

In studies without controls, Kmax and CDVA readings at baseline and at 12 months are reported in Table 2. Hernandez [23] demonstrated statistically significant improvements (*P* < 0.05) in Kmax with P-CXL at 12 months with two UV-A irradiance subgroups (30mW: -2.98 ± 3.09 D, 45mW: -2.99 ± 2.98 D). The same was also shown in CDVA for both groups (30mW: -0.06 ± 0.11 logMAR, 45mW: -0.04 ± 0.05 logMAR).

Mazzotta showed similar results in two separate studies [29, 31] with statistically significant improvements in Kmax as well as CVDA in 2017 (-1.12 ± 1.17 D; 0.12 ± 0.1 logMAR) and 2022 (-1.3 ± 0.78 D; 0.2 ± 0.08 logMAR) respectively. Gore [22] demonstrated statistical significance during 24 month follow up in Kmax readings (61.9 ± 8.1 D) and Belvirani [46] demonstrated the same for Kmax and CVDA (54.65 ± 5.36 D and 0.17 ± 0.13 logMAR respectively).

**Table 2:** Primary outcomes in pulsed cross-linking only studies with 12 month follow up (mean ± SD)

Study	Kmax baseline (D)	Kmax 12 months (D)	*p* value	CDVA baseline (logMAR)	CDVA 12 months (logMAR)	*p* value
Gaafar 2021	47.19 ± 2.63	46.91 ± 2.45	0.17	-	-	-
Hernandez 2019 (30 mW/cm^2^ group)	53.54 ± 7.20	51.09 ± 6.12	**< 0.05**	0.11 ± 0.11	0.06 ± 0.06	**< 0.05**
Hernandez 2019 (45 mW/cm^2^ group)	54.80 ± 6.66	49.98 ± 4.95	**< 0.05**	0.08 ± 0.08	0.04 ± 0.06	**< 0.05**
Mazzotta 2017	48.33 ± 2.51	47.21 ± 0.71	**0.04**	0.27 ± 0.14	0.07 ± 0.10	**< 0.05**
Mazzotta 2022	48.52 ± 1.63	47.20 ± 0.80	**< 0.05**	0.61 ± 0.19	0.80 ± 0.05	**< 0.05**
Sun 2018	54.86 ± 7.83	54.70 ± 7.86	0.94	0.28 ± 0.32	0.14 ± 0.19	**0.04**
Zhang 2020	57.29 ± 9.13	57.40 ± 8.95	0.96	0.24 ± 0.29	0.14 ± 0.22	0.23
Ziaei 2019	59.13 ± 7.40	60.08 ± 7.24	0.96	0.30 ± 0.32	0.32 ± 0.20	0.12

*CDVA= *corrected distance visual acuity

**Fig. 2:**
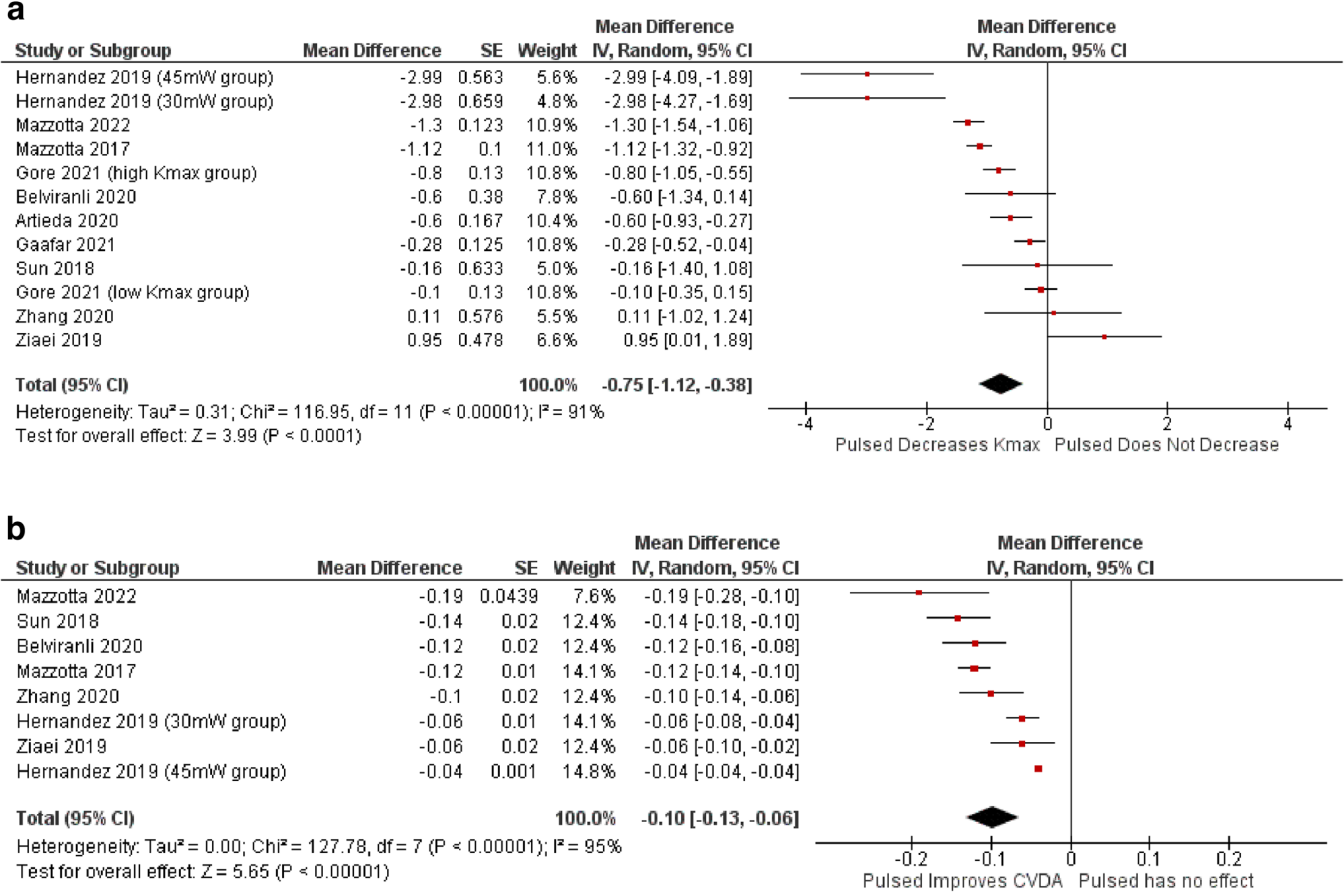
**a**. Forest plot of Kmax 12-month unadjusted mean difference in dioptres in pulsed groups in non-comparative studies included in the meta-analysis. Df = degrees of freedom; I^2^ = heterogeneity measure. **b.** Forest plot of CDVA 12-month unadjusted mean difference in logMAR in pulsed groups in non-comparative studies included in the meta-analysis. Df = degrees of freedom; I^2^ = heterogeneity measure

In total, 12 non-comparative arms from 10 studies were included in the meta-analysis. As seen in Fig. 2a, the mean difference for Kmax between groups at 12 months follow up was − 0.75 D (95% confidence interval − 1.12 to -0.38; *p* < 0.001).

Forest plot analysis for CDVA can be seen in Fig. 2b which shows a mean difference of -0.10 logMAR at 12 months (CI: -0.13 to -0.06). Similar to Kmax, a statistically significant improvement in visual acuity was noted after P-CXL treatment (*p* < 0.0001). The I^2^ statistic was 91% and 95% for Kmax and CDVA respectively.

## Comparative effectiveness of P-CXL and C-CXL

### Primary outcomes

#### Kmax

**Fig. 3:**
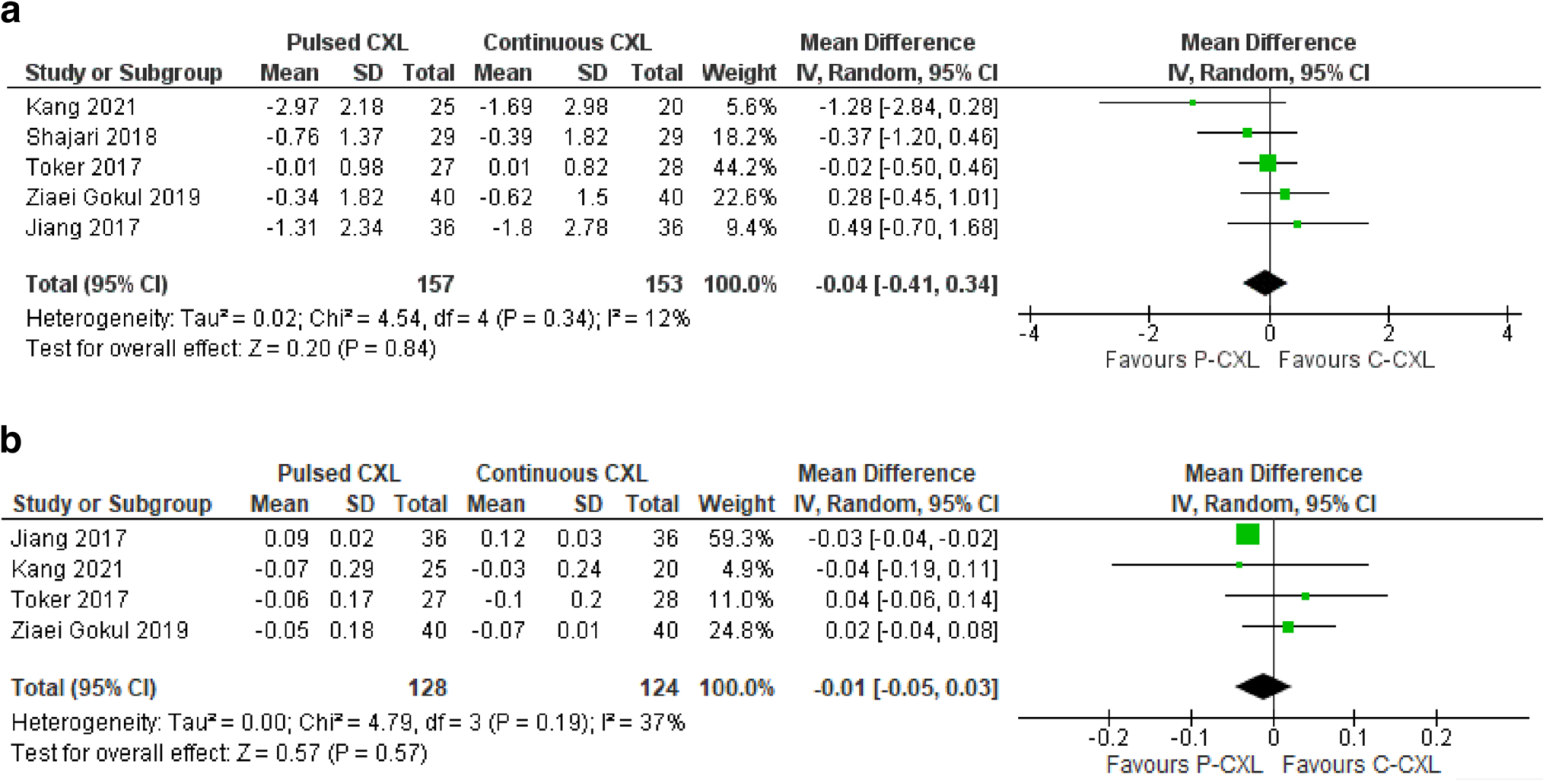
**a** Forest plot of Kmax 12-month unadjusted mean change in dioptres in pulsed and continuous groups in studies included in the meta-analysis. CXL = corneal cross-linking; df = degrees of freedom; I^2^ = heterogeneity measure. **b** Forest plot of CDVA 12-month unadjusted mean change in LogMAR in pulsed and continuous groups in studies included in the meta-analysis. CDVA = corrected distance visual acuity; CXL = corneal cross-linking; df = degrees of freedom; I^2^ = heterogeneity measure

In total, five comparative studies were included in the meta-analysis (Table 3). Based on the forest plot analysis as seen in Fig. 3a, the mean difference for Kmax between groups at 12 months follow up was − 0.04 D, with a 95% confidence interval ranging from − 0.41 to 0.34. The I^2^ statistic indicates low heterogeneity among the included studies, with only 12% of the variability attributed to true differences rather than random chance.

The narrow confidence interval indicates a relatively precise estimate of the mean difference. The small effect size suggests that CXL succeeds at stabilising Kmax at 12 months or there may be limited differences among the studies. An overall p value of 0.84 did not provide evidence of a significant mean difference in reduction of Kmax between pulsed and continuous CXL groups.

For studies not included in the meta-analysis, Marafon et al. [26] demonstrated that Kmax reduced significantly after both pulsed and continuous CXL protocols at 6 months (continuous ΔKmax − 0.68 ± 2.02, *p* = 0.016 and pulsed ΔKmax − 0.90 ± 3.12, *p* = 0.003) but with the average of eyes treated with continuous CXL lower than those treated with pulsed CXL (continuous 52.00 ± 4.91D and pulsed 54.31 ± 5.45D, *p* = 0.026). Ziaei et al. [42] reported on 24-month data for epi-off pulsed and continuous protocols at 30mW as well as another transepithelial pulsed group at 45mW (t-CXL). While all groups showed a reduction in Kmax, only the continuous CXL group showed statistical significance by the end of the observation period (baseline 57.48 ± 5.84 vs. 24 months 55.73 ± 6.04, *p* = 0.01).

### Visual acuity

Marafon et al. [26] demonstrated that CDVA improved significantly in both pulsed and continuous CXL protocols at 6 months (continuous ΔCDVA − 0.04 ± 0.14, *p* = 0.016 and pulsed ΔCDVA − 0.10 ± 0.2, *p* = 0.001). On multiple regression, they found that the associations between post-operative CDVA with baseline CDVA or Kmax were not statistically significant (both *p* > 0.05). Ziaei et al. [42] similarly demonstrated statistically significant improvements in CDVA in all three arms – continuous (baseline: 0.36 ± 0.22 vs. 24 month: 0.26 ± 0.27, *p* = 0.02) pulsed 30mW (baseline: 0.30 ± 0.16 vs. 24 month: 0.23 ± 0.17, *p* = 0.04) and pulsed 45mW (baseline: 0.38 ± 0.32 vs. 24 month: 0.30 ± 0.21, *p* = 0.04). A study by Mazzotta et. al [28] did not present visual acuity data in logMAR form but data showed non-statistically significant improvements in CDVA in both pulsed (1.8 ± 1.3 decimal equivalents, *p* = 0.55) and continuous groups (+ 1.6 ± 1.0 decimal equivalents, *p* = 0.56).

Table 2 demonstrates that P-CXL is statistically significant in improving visual outcomes in 12 months and there is no significant difference between P-CXL and C-CXL modalities. A meta-analysis for CDVA (Fig. 3b) included four studies showing a -0.01 logMAR mean change between pulsed and continuous groups at 12 months (CI, -0.05 to 0.03; *p* = 0.57). A moderate heterogeneity (I^2^ = 37%) suggests some variability in effect sizes observed. Consistent data is presented in the case of uncorrected distance visual acuity (UDVA) (Online Resource 2).

### Secondary Outcomes

#### Kmean

Mazzotta et al [28] demonstrated a statistically significant decrease of topographical Kmean one year after P-CXL by a mean value − 1.2 ± 0.13 dioptres (*p* = 0.049), as compared to the C-CXL group − 0.13 ± 0.13 (*p* = 0.088). Comparative studies [26, 42] assessing pulsed and continuous CXL at 6 or 24 months respectively, showed no statistically significant differences in the reduction of Kmean between groups.

Three studies were included in the meta-analysis for Kmean; mean change in Kmean between P-CXL and C-CXL groups after 12 months was − 0.30 D (CI, -0.66 to 0.06; *p* = 0.11). The heterogeneity was moderate (I2 = 34%) between studies (Online Resource 3).

**Table 3:** Primary outcomes in included pulsed vs. continuous studies with 12 month follow up (mean ± SD)

Study	Arms	Kmax baseline (D)	ΔKmax at 12 months (D)	*p* value	CDVA baseline (logMAR)	ΔCDVA at 12 months (logMAR)	*p* value
Jiang 2017	Pulsed group (*n* = 36)	53.05 ± 4.80	-1.31 ± 2.34	**< 0.05**	0.28 ± 0.23	0.09 ± 0.02	**< 0.05**
	Continuous group (*n* = 36)	54.38 ± 5.65	-1.80 ± 2.78	**< 0.05**	0.36 ± 0.25	0.12 ± 0.03	**< 0.05**
Kang 2021	Pulsed group (*n* = 25)	57.10 ± 7.06	-2.97 ± 2.18	**< 0.05**	0.40 ± 0.43	-0.07 ± 0.29	0.28
	Continuous group (*n* = 20)	54.30 ± 9.41	-1.69 ± 2.98	**< 0.05**	0.33 ± 0.37	0.30 ± 0.34	0.55
Shajari 2018	Pulsed group (*n* = 29)	55.20 ± 8.10	-0.76 ± 1.37	0.26	-	-	-
	Continuous group (*n* = 29)	56.20 ± 8.60	-0.39 ± 1.82	1	-	-	-
Toker 2017	Pulsed group (*n* = 27)	56.68 ± 6.10	-0.01 ± 0.98	**> 0.05**	0.27 ± 0.22	-0.06 ± 0.17	**> 0.05**
	Continuous group (*n* = 28)	56.61 ± 6.10	0.01 ± 0.82	**> 0.05**	0.32 ± 0.26	-0.10 ± 0.2	**0.02**
Ziaei, Gokul 2019	Pulsed group (*n* = 40)	58.11 ± 5.60	−0.34 ± 1.82	0.47	0.30 ± 0.16	−0.05 ± 0.18	0.27
	Continuous group (*n* = 40)	57.48 ± 5.84	−0.62 ± 1.50	0.56	0.36 ± 0.22	−0.07 ± 0.01	**0.07**

*CDVA= *corrected distance visual acuity

### Pachymetry (central corneal thickness)

Three studies were included in the meta-analysis for CCT; mean change in CCT between P-CXL and C-CXL groups after 12 months was − 1.44 μm (CI, -12.12 to 9.25; *p* = 0.79). Forest plot showed heterogeneity was high (I^2^ = 82%) between studies (Online Resource 4).

### Demarcation line

A study by Peyman et al. [34] revealed stromal demarcation depth detected by anterior segment optical coherence tomography (OCT) was statistically deeper (*p* < 0.001) for the pulsed group measuring 201.11 ± 27.76 μm vs. 159.88 ± 20.86 μm for the continuous group at 3 months. This was similarly demonstrated by Moramarco [32] (P-CXL: 213 ± 47.38 μm vs. C-CXL: 149.32 ± 36.03 μm; *p* < 0.001) and Mazzotta [28] (P-CXL: 215 ± 20 μm vs. C-CXL: 160 ± 20 μm; *p* < 0.001) at 1 month. A study by Kang [25] (P-CXL: 191.6 ± 30.3 μm vs. C-CXL: 160.9 ± 20.6 μm, *p* = 0.02) showed statistically deeper demarcation lines at 12 months.

### Effectiveness of P-CXL with modifications

#### Topographically guided P-CXL – photoreactive intrastromal crosslinking (PiXL)

A randomised control trial conducted by Nordstrom [33] comparing P-CXL with PiXL in 50 eyes found that mean reduction in Kmax (− 1.31 ± 1.52 D), CDVA (− 0.16 ± 0.24 logMAR) and UDVA (− 0.31 ± 0.40 logMAR) at 12 months was statistically significant only in the topographically guided pulsed (TGP-CXL) group (*p* < 0.01). Eyes randomly allocated to PiXL were treated with varied levels of fluence (either 7.2 J/cm^2^, 10 J/cm^2^, or 15 J/cm^2^) depending on the grading of their disease. Those that received 15 J/cm^2^ showed a larger reduction in Kmax at 12 months as compared to eyes treated with 7.2–10 J/cm^2^ (− 1.74 ± 1.66 D vs. − 0.40 ± 0.53 D; *p* = 0.01). In a prospective cohort study by Cassagne [20] comparing PiXL with C-CXL, a significant decrease of Kmax was observed in the PiXL group at 12 months (-1.07 ± 1.70, *p* < 0.001), with no significant changes in C-CXL eyes (0.40 ± 1.75 D, *p* = 0.26), representing a statistically significant difference between the two groups (*p* < 0.01). At 12 months, CDVA and UDVA improved to 0.22 ± 0.25 (*p* < 0.05) and 0.65 ± 0.40 (*p* = 0.56) logMAR respectively, in the PiXL group and 0.26 ± 0.26 (*p* = 0.10) and 0.61 ± 0.35 (*p* = 0.20) logMAR, respectively, in the C-CXL group. Sixty-four eyes undergoing either PiXL or C-CXL in a prospective study by Sachdev [35] found a significant reduction in Kmax in the PiXL group at 12 months (-1.6 ± 1.79 D, *p* = 0.001). The CDVA improved significantly in the PiXL group (0.05 ± 0.08 logMAR, *p* = 0.02) versus the C-CXL (0.01 ± 0.03 logMAR, *p* = 0.26) group.

#### P-CXL delivery aided with supplemental oxygen

In a non-comparative study by Matthys [27], 34 eyes received P-CXL accompanied by goggles delivering high levels of oxygen. At 12 months, mean Kmax was reduced by -1.56 ± 1.71 D (*p* < 0.0001) and mean CDVA improved by 0.093 ± 0.193 logMAR (*p* < 0.02). The mean Kmean decreased by 0.40 ± 0.78 D (*p* < 0.01) and there were no changes in UDVA, pachymetry or endothelial cell count (all *p* > 0.05). Another study [19] utilising oxygen goggles and higher fluence (30mW/cm^2^) on 53 eyes noted no statistically significant changes in Kmax or Kmean but observed a statistically significant improvement in CDVA from 0.18 ± 0.21 logMAR at baseline to 0.07 ± 0.15 logMAR at 12 months (*p* < 0.01). A study by Mazzotta [30] utilising oxygen goggles with P-CXL in 27 eyes showed significant improvement of Kmax at 6 months (-1.91 ± 1.62 D, *p* < 0.05) as well as CDVA (-0.08 ± 0.03 logMAR, *p* < 0.05).

## Discussion

This systematic review and meta-analysis evaluated the effectiveness of P-CXL in the treatment of keratoconus and assessed whether P-CXL had superior outcomes compared to C-CXL modalities included 2866 eyes (P-CXL 1817; C-CXL 1049) from 29 publications. Studies included were primarily retrospective and prospective cohort studies or case series in addition to one randomised control trial. For the meta-analysis, we found ten studies [17, 21–23, 29, 31, 37, 40, 41, 46] assessing the efficacy of P-CXL in keratoconus and five [24, 25, 36, 39, 43] studies that directly compared patient groups receiving pulsed or continuous CXL reporting our outcomes of interest at 12 months follow up. These studies were ascertained to be satisfactory or good in a quality assessment based on the Newcastle Ottawa Scale (see Appendix).

Overall, P-CXL is effective in improving visual acuity and keratometry outcomes in keratoconus as demonstrated by the meta-analysis for single arm studies (see Fig. 2a and b). At 12 months P-CXL showed statistically significant reductions in Kmax (*p* < 0.001) and CDVA (*p* < 0.001) compared to baseline. The I^2^ statistics for both (91% and 95%) indicate a high level of heterogeneity, attributed to varying UV-A energy levels, patient grading of keratoconus (high or low kmax groups), ethnicity and other factors that would require further subgroup analysis or meta regression analyses.

The meta-analysis of comparative studies determined that mean differences in Kmax, CDVA, UDVA, Kmean and CCT after 12 months were not statistically significant between pulsed and continuous crosslinking groups. Thus, pooled results suggest that both modalities of crosslinking procedures are comparable in stabilising vision and keratometry in keratoconus. It is noted that Kmax was chosen as a primary outcome measure due to common use in literature. Ideally, zonal parameters should be used as it demonstrates more reliable measurement repeatability.

While further data on corneal endothelial morphometry and higher order aberrations could paint a broader picture in the overall assessment of efficacy, studies within the meta-analysis did not report on these outcomes consistently. As these outcomes are not routinely evaluated in clinical practice, our review may still be considered comprehensive despite not assessing these outcomes.

Remaining studies included in the systematic review but not the meta-analysis added valuable insight as they assessed the efficacy of P-CXL with modifications, looked at secondary outcomes or at time points other than 12 months. Secondary outcomes assessed included demarcation line. A demarcation line following a CXL procedure describes the depth of penetration into the corneal stroma, highlighting a transition zone between the treated anterior and untreated posterior layers and is theoretically correlated with the overall efficacy of treatment. Studies [32, 34] that demonstrated deeper demarcation lines as a result of P-CXL, as well as evidence displayed in animal studies [47] showed P-CXL to be less injurious and more efficacious than C-CXL lend credence to the suggestion that P-CXL may result in superior procedural and treatment outcomes clinically. In practice, the basis of evidence for the superiority of P-CXL still needs to be established to provide paradigm shifts in clinical decision making to support P-CXL as the new standard of care.

For studies assessing P-CXL with modifications, the use of supplemental oxygen via goggles demonstrated statistically significant outcomes in Kmax (*P* < 0.01) [27] as well as CVDA (*P* < 0.01) [19] which is consistent with meta-analyses conducted on the subject [48]. In studies evaluating PiXL [20, 33, 35], fluence levels tended to be higher (up to 15 J/cm^2^) but all studies reviewed showed superiority in primary outcomes in PiXL groups as compared to C-CXL, suggesting that topographically guided CXL is a promising avenue in the evolution of progressive keratoconus treatment.

Endothelial cell density is an important factor in safety as it has implications for long-term safety. Endothelial cell count was assessed in several studies [16, 20, 35] where P-CXL mostly showed a non-significant reduction in cell density, indicating minimal endothelial damage and overall stability of the cells. No significant changes in endothelial cell density were noted between P-CXL and C-CXL groups in studies. While it is generally considered that stromal demarcation lines are deeper in P-CXL, the relevance is debated and studies must assess clinically relevant parameters to ensure there is a real benefit to patients of a treatment. There remains some controversy, with some studies [32] arguing that it is no longer detectable after six months, while others [25, 28, 39] demonstrate that demarcation is visible up until 12 months. It is noted that deeper lines may be attributed to the longer treatment times that result from intermittent pulses.

Safety data among groups were not formally assessed in any of the studies. The only mention of safety outcomes noted patients that experienced common adverse events equally distributed among groups [24] (such as corneal haze) or that patients did not respond to treatment and saw progression of their keratoconus [21].

The 12-month timeframe chosen for the examination of data within the meta-analysis represents a relatively brief follow-up period for the chronic nature of keratoconus [49, 50]. As a condition with a prolonged course, longer-term data would have been preferred if consistent follow up data were available. Despite this, clinical decision-making surrounding keratoconus treatment is frequently made within shorter time frames as optimal outcomes in arresting ectasia are best achieved with swift and early intervention [51, 52].

There remains a paucity in the number of randomised control trials conducted involving CXL. As such, this systematic review and meta-analysis comprised mainly non-randomised studies that utilise less robust methods of appraisal for risk of bias and quality. In addition, non-randomised studies tend to exhibit greater heterogeneity as evidenced by the I^2^ statistic in outcomes analysed – Kmax (12%), CDVA (37%), Kmean (34%), CCT (82%). The nonrandomised studies showed deficiencies in addressing confounding factors and describing subgroups, most notably lack of controls for a patient’s disease stage which can impact their response to treatment [53]. A total fluence level of 5.4 J/cm^2^ is outlined by the original Dresden protocol and utilised in most clinical settings. Despite a fluence level of 5.4 J/cm^2^ remaining gold standard in clinical practice, a fluence level of 7.2 J/cm^2^ was used in the published reports as it is thought that higher fluences increase the efficiency of the treatment [30] and is the setting available on most CXL machines. Future analyses comparing P-CXL and C-CXL may benefit from selecting studies with identical energy levels and time exposures as compared to fluence level for a more robust comparison.

Another limitation was the lack of data available for statistical mixed methods models that may account for variations in study design among papers that skew the results of the meta-analysis. These variations may include protocols on epithelial debridement, how to measure key tomographic indices or the presence of contact lenses and their removal. All these factors consequently limit the reliability of the conclusions drawn from the meta-analysis.

In conclusion, this systematic review and meta-analysis demonstrated that P-CXL is efficacious in the treatment of keratoconus, with pulsed and continuous CXL modalities displaying no statistical significance between efficacy outcomes in keratometry, visual acuity or pachymetry. Studies performed were heterogeneous and bias was present in confounding factors such as variability in ethnicity affecting patient response and methodology by which statistical analyses were conducted. Other bias was present regarding data loss to follow up as well as selection bias with mostly single centre limitations. Further multicentre randomised studies are needed with better descriptions of how outcomes were measured and analysed to provide higher quality evidence.

## Electronic supplementary material

Below is the link to the electronic supplementary material.

Supplementary Material 1
