# Supplementary material for: Pulsed corneal crosslinking in the treatment of Keratoconus: a systematic review and meta-analysis
Source: Graefes Arch Clin Exp Ophthalmol. 2024 Aug 31;263(3):589–601. doi: 10.1007/s00417-024-06622-7 (PMC11953132; doi:10.1007/s00417-024-06622-7)
Supplement: Supplementary file 1 — Supplementary Material 1 [file 417_2024_6622_MOESM1_ESM.pdf]

Supplementary Information

Title: Pulsed Corneal Crosslinking in the Treatment of Keratoconus: A Systematic Review and Meta-Analysis

Journal: Graefe's Archive for Clinical and Experimental Ophthalmology

Authors: Maria Qureshi[1], Stephanie L Watson OAM*, MBBS, PhD, FRANZCO[1], Himal Kandel*, PhD[1]

1. The University of Sydney, Faculty of Medicine and Health, Save Sight Institute, Sydney, NSW, Australia *Joint senior authors

Correspondence: Professor Stephanie L Watson OAM, the University of Sydney, Save Sight Institute, Sydney Eye Hospital, South Block, Level 1, 8 Macquarie Street, NSW 2000, Australia. Email: stephanie.watson@sydney.edu.au

Online Resource 1

	# ▲	Searches	Results	Type	Actions	Annotations
☐	1	keratoconus.mp. or Keratoconus/	8461	Advanced	Display Results | More ▾	
☐	2	*eye diseases/ or dilatation, pathologic/	38747	Advanced	Display Results | More ▾	
☐	3	ectasia.mp.	5682	Advanced	Display Results | More ▾	
☐	4	1 or 2 or 3	50388	Advanced	Display Results | More ▾	
☐	5	Corneal Stroma/ or Cross-Linking Reagents/ or Ultraviolet Rays/ or Photochemotherapy/ or Cornea/ or Riboflavin/ or Keratoconus/ or corneal cross linking.mp. or Photosensitizing Agents/	197490	Advanced	Display Results | More ▾	
☐	6	cxl.mp.	1579	Advanced	Display Results | More ▾	
☐	7	5 or 6	197780	Advanced	Display Results | More ▾	
☐	8	pulsed.mp.	74357	Advanced	Display Results | More ▾	
☐	9	intermittent.mp.	98238	Advanced	Display Results | More ▾	
☐	10	8 or 9	172244	Advanced	Display Results | More ▾	
☐	11	4 and 7 and 10	49	Advanced	Display Results | More ▾	

Supplementary Figure 1. Search strategy for the systematic review and meta-analysis of pulsed vs continuous crosslinking

Online Resource 2

Study or Subgroup	Pulsed CXL Mean	Pulsed CXL SD	Pulsed CXL Total	Continuous CXL Mean	Continuous CXL SD	Continuous CXL Total	Weight	Mean Difference IV, Random, 95% CI
Jiang 2017	0.12	0.04	36	0.14	0.05	36	59.6%	-0.02 [-0.04, 0.00]
Kang 2021	-0.04	0.37	25	-0.22	0.54	20	1.3%	0.18 [-0.10, 0.46]
Toker 2017	0.02	0.25	27	-0.05	0.17	28	7.4%	0.07 [-0.04, 0.18]
Ziaei Gokul 2019	-0.04	0.12	40	-0.02	0.08	40	31.6%	-0.02 [-0.06, 0.02]
**Total (95% CI)**			**128**			**124**	**100.0%**	**-0.01 [-0.04, 0.02]**

Heterogeneity: Tau² = 0.00; Chi² = 4.29, df = 3 (P = 0.23); I² = 30%
Test for overall effect: Z = 0.64 (P = 0.52)

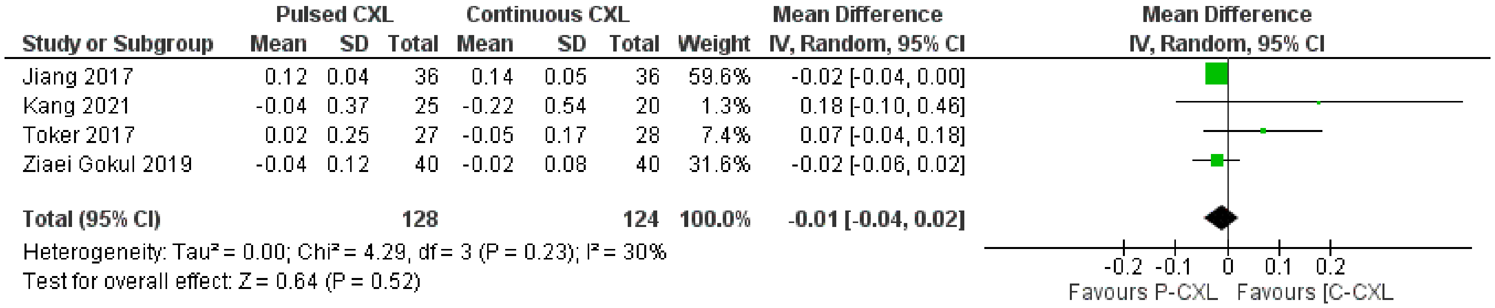

Supplementary Figure 2. Forest plot of UDVA 12-month unadjusted mean change in LogMAR in pulsed and continuous groups in studies included in the meta-analysis. df = degrees of freedom; I^2 = heterogeneity measure.

Online Resource 3

Study or Subgroup	Pulsed CXL Mean	Pulsed CXL SD	Pulsed CXL Total	Continuous CXL Mean	Continuous CXL SD	Continuous CXL Total	Weight	Mean Difference IV, Random, 95% CI
Kang 2021	-1.68	1.46	25	-0.87	0.9	20	21.0%	-0.81 [-1.51, -0.11]
Toker 2017	-0.08	0.59	27	0.15	0.52	28	56.3%	-0.23 [-0.52, 0.06]
Ziaei Gokul 2019	0.02	1.24	40	0.02	1.73	40	22.7%	0.00 [-0.66, 0.66]
**Total (95% CI)**			**92**			**88**	**100.0%**	**-0.30 [-0.66, 0.06]**

Heterogeneity: Tau² = 0.04; Chi² = 3.03, df = 2 (P = 0.22); I² = 34%
Test for overall effect: Z = 1.61 (P = 0.11)

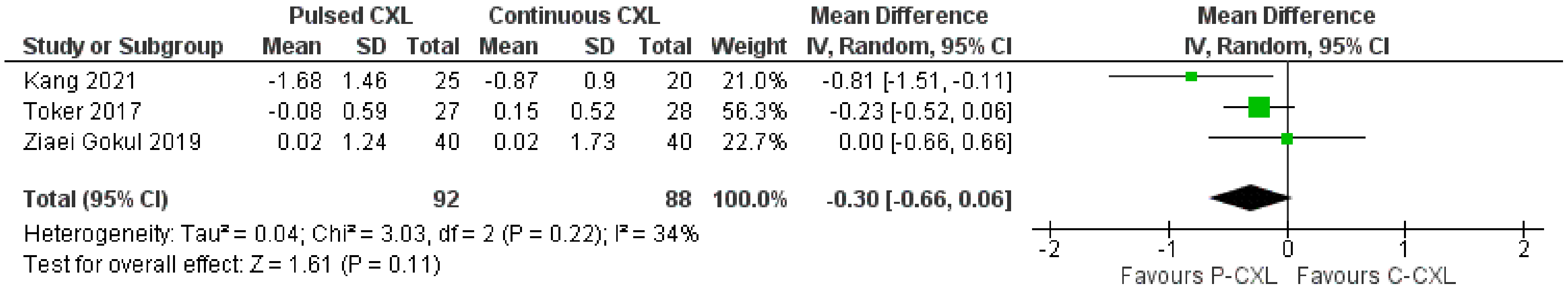

Supplementary Figure 3. Forest plot of Kmean 12-month unadjusted mean difference in dioptres in pulsed and continuous groups in studies included in the meta-analysis. df = degrees of freedom; I^2 = heterogeneity measure.

Online Resource 4

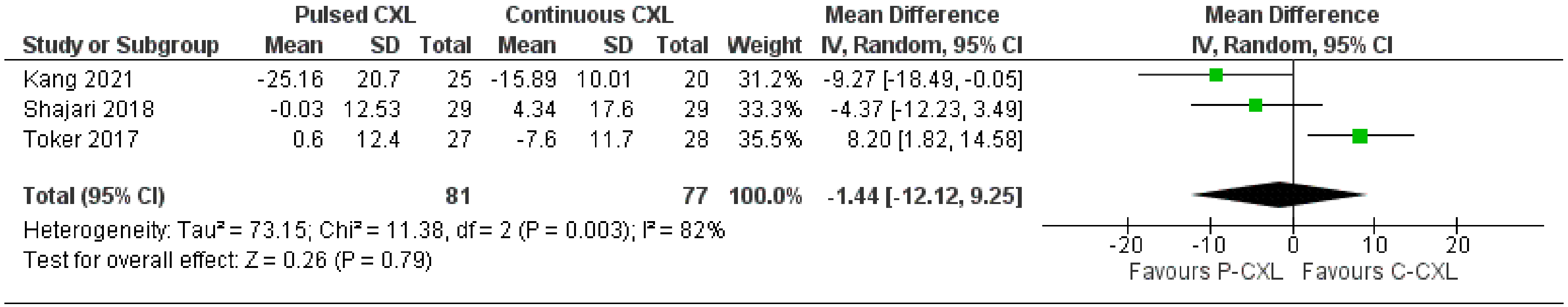

Supplementary Figure 4. Forest plot of CCT 12-month unadjusted mean difference in µm in pulsed and continuous groups in studies included in the meta-analysis. df = degrees of freedom; I^2 = heterogeneity measure.

Online Resource 5

Supplementary Table 1. The Newcastle-Ottawa Scale (NOS) quality assessment of the included case control and cohort studies for the systematic review and meta-analysis of pulsed vs continuous crosslinking.[46]

Case Control Studies

**Study**	**Selection of cases and controls**				**Comparability of cases and controls**	**Exposure**			**Total Score (stars)**
	Is the case definition adequate?	Representativeness of the cases	Selection of controls	Definition of controls	Comparability of cases and controls on the basis of design or analysis	Ascertainment of CXL procedure	Same method of ascertainment for cases and controls?	Non-response Rate	
Shajari 2018	a*	b	a*	a*	a*	a*	a*	a*	7
Toker 2017	a*	b	a*	a*	a*	a*	a*	b	6
Ziaei, Gokul 2019	a*	b	a*	b	a*	a*	a*	a*	6

Cohort Studies

Study	Selection of Cohorts				Comparability of cases and controls	Outcome			Total Score (stars)
	Representativeness of the pulsed CXL cohort	Selection of the continuous CXL cohort	Ascertainment of CXL procedure	Demonstration that outcome of interest was not present at start of study	Comparability of cohorts on the basis of design or analysis	Ascertainment of outcomes	Was follow up long enough for outcomes to occur	Adequacy of follow up of cohorts	
Jiang 2017	b	a*	a*	a*	a*	a*	a*	a*	7
Kang 2021	b	a*	a*	a*	a*	a*	a*	b	6

Online Resource 6

Supplementary Table 2. The Murad et. al quality assessment of the included case series for the systematic review and meta-analysis of pulsed vs continuous crosslinking.[13]

Non-Comparative Case Series

	**Domains**	**Ascertainment**		**Causality**		**Reporting**	
**Study**	Do the patients represent the whole experience of the investigator (centre) or is the selection method unclear to the extent that other patients with similar presentation may not have been reported?	Was the CXL procedure type adequately ascertained?	Were the outcomes (Kmax, CDVA, etc) adequately ascertained?	Were other alternative causes that may explain the observation ruled out?	Was follow-up long enough (>12 months) for outcomes to occur?	Are the cases described with sufficient details to allow other investigators to replicate the research or to allow practitioners to make inferences related to their own practice?	**Overall Quality**
Artieda 2020	Yes	Yes	Yes	Yes	Yes	Yes	Good
Belviranli 2020	Yes	Yes	Yes	Yes	Yes	Yes	Good
Gaafar 2021	Yes	Yes	Yes	Yes	Yes	Yes	Good
Gore 2021	Yes	Yes	Yes	Yes	Yes	Yes	Good

Hernandez 2019	Yes	Yes	Yes	Yes	Yes	Yes	Good
Mazzotta 2017	No*	Yes	Yes	Yes	Yes	Yes	Good
Mazzotta 2022	Yes	Yes	Yes	Yes	Yes	Yes	Good
Sun 2018	Yes	Yes	Yes	Yes	Yes	Yes	Good
Zhang 2020	Yes	Yes	Yes	Yes	Yes	Yes	Good
Ziaei 2019	Yes	Yes	Yes	Yes	Yes	Yes	Good

***Included only Grade II Keratoconus patient**

Newcastle/Ottawa Quality Assessment Scale – Case Control Studies[46]

Selection

1) Is the case definition adequate?

a) yes, with independent validation*

b) yes, eg record linkage or based on self reports

c) no description

2) Representativeness of the cases

a) consecutive or obviously representative series of cases*

b) potential for selection biases or not stated

3) Selection of Controls

a) drawn from the same community as the case cohort*

b) drawn from a different source

c) no description of the derivation of controls

4) Definition of Controls

a) no history of CXL*

b) no description of source

Comparability

1) Comparability of cases and controls on the basis of the design or analysis

a) study controls for age and gender*

b) study controls for additional factors (contact lens wearers, ethnicity, etc)*

Exposure

1) Ascertainment of exposure

a) secure record (eg surgical records)*

b) structured interview where blind to case/control status

c) interview not blinded to case/control status

d) written self report or medical record only

e) no description

2) Same method of ascertainment for cases and controls

a) yes*

b) no

3) Non-Response rate

a) same rate for both groups*

b) non respondents described

c) rate different and no designation

Scoring

Good studies (7-9 stars)

Satisfactory studies (5-6 stars)

Poor studies (<4 stars)

Newcastle/Ottawa Quality Assessment Scale – Cohort Studies[46]

Selection

1) Representativeness of the pulsed CXL cohort

a) truly representative of the average participants in the community*

b) somewhat representative of the average participants in the community

c) selected group of users eg nurses, volunteers

d) no description of the derivation of the cohort

2) Selection of the continuous CXL cohort

a) drawn from the same community as the pulsed CXL cohort*

b) drawn from a different source

c) no description of the derivation of the continuous CXL cohort

3) Ascertainment of CXL procedure

a) secure record (eg surgical records)*

b) structured interview

c) written self report

d) no description

4) Demonstration that outcome of interest was not present at start of study

a) yes*

b) no

Comparability

1) Comparability of cohorts on the basis of the design or analysis

a) study controls for age and gender*

b) study controls for additional factors (contact lens wearers, ethnicity, etc)*

Outcome

1) Assessment of outcome

a) independent blind assessment*

b) record linkage

c) self report

d) no description

2) Was follow-up long enough for outcomes to occur (>12 months)?

a) yes*

b) no

3) Adequacy of follow up of cohorts

a) complete follow up - all subjects accounted for*

b) subjects lost to follow up unlikely to introduce bias - small number lost (<10 % follow up, or description provided of those lost)

c) follow up rate < 75% and no description of those lost

d) no statement

Scoring

Good studies (7-9 stars)

Satisfactory studies (5-6 stars)

Poor studies (<4 stars)
